# First-time use of electronic patient-reported outcome measures in a cluster randomized trial: a qualitative study

**DOI:** 10.1186/s41687-025-00840-1

**Published:** 2025-01-20

**Authors:** Terese Solvoll Skåre, Tonje Lundeby, Jo Åsmund Lund, Marianne Jensen Hjermstad, May Helen Midtbust

**Affiliations:** 1 Department of Health Sciences Ålesund, Faculty of Medicine and Health Sciences, Norwegian University of Science and Technology, Ålesund, Norway; 2 Regional Advisory Unit for Palliative Care, Department of Oncology, Oslo University Hospital,, Oslo, Norway; 3 Department of Oncology, European Palliative Care Research Centre (PRC), Oslo University Hospital, Institute of Clinical Medicine, University of Oslo, Oslo, Norway; 4 Clinic for Cancer Treatment and Rehabilitation, Møre og Romsdal Hospital Trust, Ålesund, Norway

**Keywords:** Electronic patient-reported outcome measures, Oncology care, Implementation science, Symptom management, Health care professionals, Qualitative research

## Abstract

**Background:**

Although there is clear evidence supporting the beneficial effects of regularly assessing patient-reported outcomes (PROs), the comprehensive integration of patient-reported outcome measures (PROMs) into routine cancer care remains limited. This study aimed to explore the facilitators and barriers encountered by principal investigators (PIs) (oncologists) and study nurses during the implementation of the Eir ePROM within a cluster randomized trial (c-RCT) in cancer outpatient clinics. Additionally, we sought to examine the influence of Eir on the working routines of the participants.

**Methods:**

Individual semi-structured interviews and a focus group were conducted with nine oncologists and study nurses involved in the implementation of the ePROM tool Eir. Interviews elucidated their experiences of barriers and facilitators when implementing Eir through a cluster randomized trial. Data were analysed according to Framework Analysis, using both an inductive and deductive approach. The Consolidated Framework for Implementation Research (CFIR) was used in the deductive stages of analysis.

**Results:**

Three overarching themes were identified from the data: (1) Willingness to invest; accepting that new eras come with a cost, (2) Management anchoring; changes start at the top, and (3) Creation of a cohesive framework; fostering collective comprehension. We found a notable disparity between oncologists and nurses in their willingness to invest time and effort in implementing the tool. While participants recognized the need to transform patient consultation methods to benefit from digital symptom management, opinions varied on whether the potential benefits justified the associated cost. Furthermore, the degree of management anchoring at various levels significantly impacted the implementation process. At the local level, it was seen as either a facilitator or a barrier, influencing the outcome of the implementation. Additionally, establishing a cohesive framework was crucial, as this fostered a collective understanding among those involved in the implementation.

**Conclusions:**

Our study underscores the importance of considering the diverse perspectives of health care professionals and fostering interprofessional collaboration for the successful implementation of ePROMs in healthcare settings. Future research should explore strategies to bridge professional disparities and promote a shared understanding of the value provided by ePROMs.

**Supplementary Information:**

The online version contains supplementary material available at 10.1186/s41687-025-00840-1.

## Introduction

### Background

Despite the increasing incidence of cancer, the predominant focus in research and treatment continues to be a tumour-centred approach. This focus remains even for patients at the end-of-life stage, despite the marginal impact of such treatment [1]. Patients with advanced cancer often bear a significant symptom burden, which profoundly affects their quality of life (QoL) and overall well-being [2, 3]. Despite the extensive knowledge about these symptoms, they are frequently underreported, undetected, or underestimated by healthcare professionals (HCPs) [4]. Therefore, systematic symptom assessment is crucial for patients with advanced cancer [5, 6].

Numerous randomized studies have documented the manifold benefits of integrating palliative care (PC) with anticancer treatment. These benefits include improved symptom management and QoL, increased satisfaction with care, and even the potential for prolonged survival [7–9]. The cornerstone of PC is a patient-centred approach that focuses on the patient with the disease, not solely the tumour [8], thereby facilitating a holistic approach. This approach aligns with the recommendations from both the American Society of Clinical Oncology (ASCO) and European Society for Medical Oncology (ESMO), which underscore the importance of integrating patient-centred care (PCC) into clinical cancer care [5, 10].

Patient-reported outcome measures (PROMs) are widely recognized as the gold standard for the efficient evaluation of patients’ perception of their own health and well-being [5, 11, 12]. PROMs enhance PCC by acknowledging patients’ assessments of their symptoms, functioning, and well-being, as well as their care preferences. Moreover, PROMs facilitate the improvement of symptom control and health-related outcomes [13], the enhancement of patient-clinician communication [14], and the promotion of patient self-management and self-efficacy [15, 16]. This recognition not only improves health outcomes but also fosters patient engagement and facilitates shared decision-making processes [5, 13, 17]. In clinical research, PROMs are extensively utilized to measure outcomes across diverse populations and evaluate the quality of care [11]. However, integrating PROMs into routine clinical practice has proven to be considerably more challenging.

Historically, the acquisition of PROMs has been primarily facilitated through conventional methods such as pen-and-paper or through informal dialogues. Over the past two decades, numerous electronic PROMs (ePROMs) have been developed, offering unparalleled opportunities to gather real-time information directly from patients using mobile phones, tablets, or computers. Furthermore, ePROMs can enhance data-quality, prove cost-effective, reduce administrative times, and facilitate similar or faster completion times [13, 14, 18].

To harness the potential benefits, ePROMs must be integrated into daily clinical cancer care. A cross-study analysis has revealed that barriers to the systematic use of PROMs/ePROMs are consistent across various settings and populations. However, facilitators are context-specific for each implementation [19]. Barriers exist at multiple levels, including contextual, institutional, and organizational. These barriers encompass inadequate stakeholder engagement, resistance to change, and competing interests within existing workflows [18]. To surmount these obstacles, it is imperative to identify both general and local factors influencing the uptake and implementation of an intervention [20]. An in-depth evaluation in the later phases of implementation can offer insight into the clinical processes involved in implementing ePROMs, as well as the impact on workflows supported by the intervention [19, 20].

### Objective

The primary objective of this study was to explore the facilitators and barriers encountered by principal investigators (PIs) (oncologists) and study nurses during the implementation of the Eir ePROM within a cluster randomized trial (c-RCT) in cancer outpatient clinics. Additionally, we sought to examine the influence of Eir on the working routines of the participants.

## Methods

### Study design

This study utilized a qualitative research design to delve into the experiences of HCPs during the implementation of the ePROM, “Eir”, as part of a c-RCT. The research method employed included semi-structured individual interviews and a focus group (FG), which was conducted following the inclusion of patients in the PALLiON trial (Palliative Care Integrated in Oncology) [21].

### Eir

**Fig. 1:**
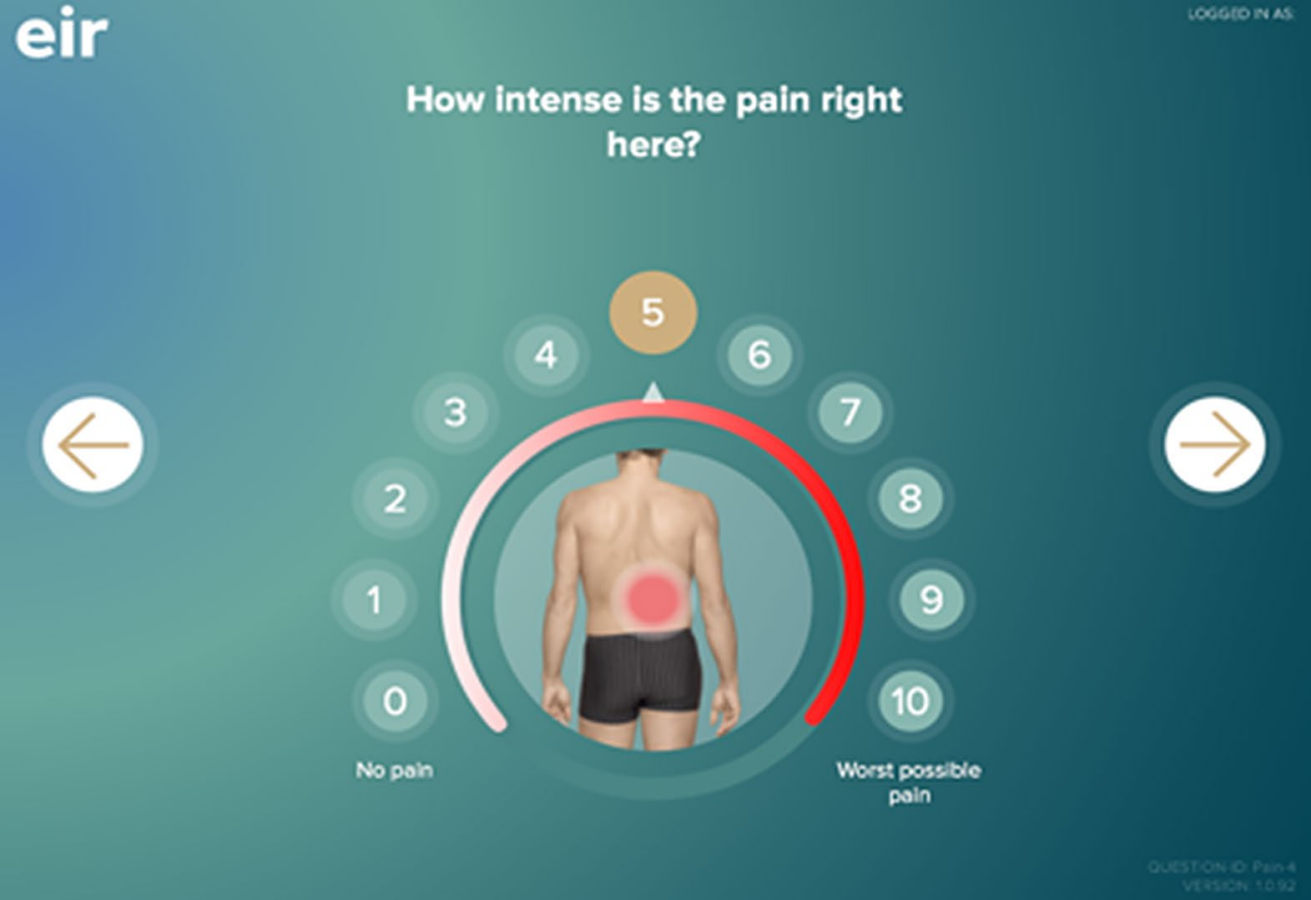
Eir-patient: symptom intensity for pain including body map [22]

**Fig. 2:**
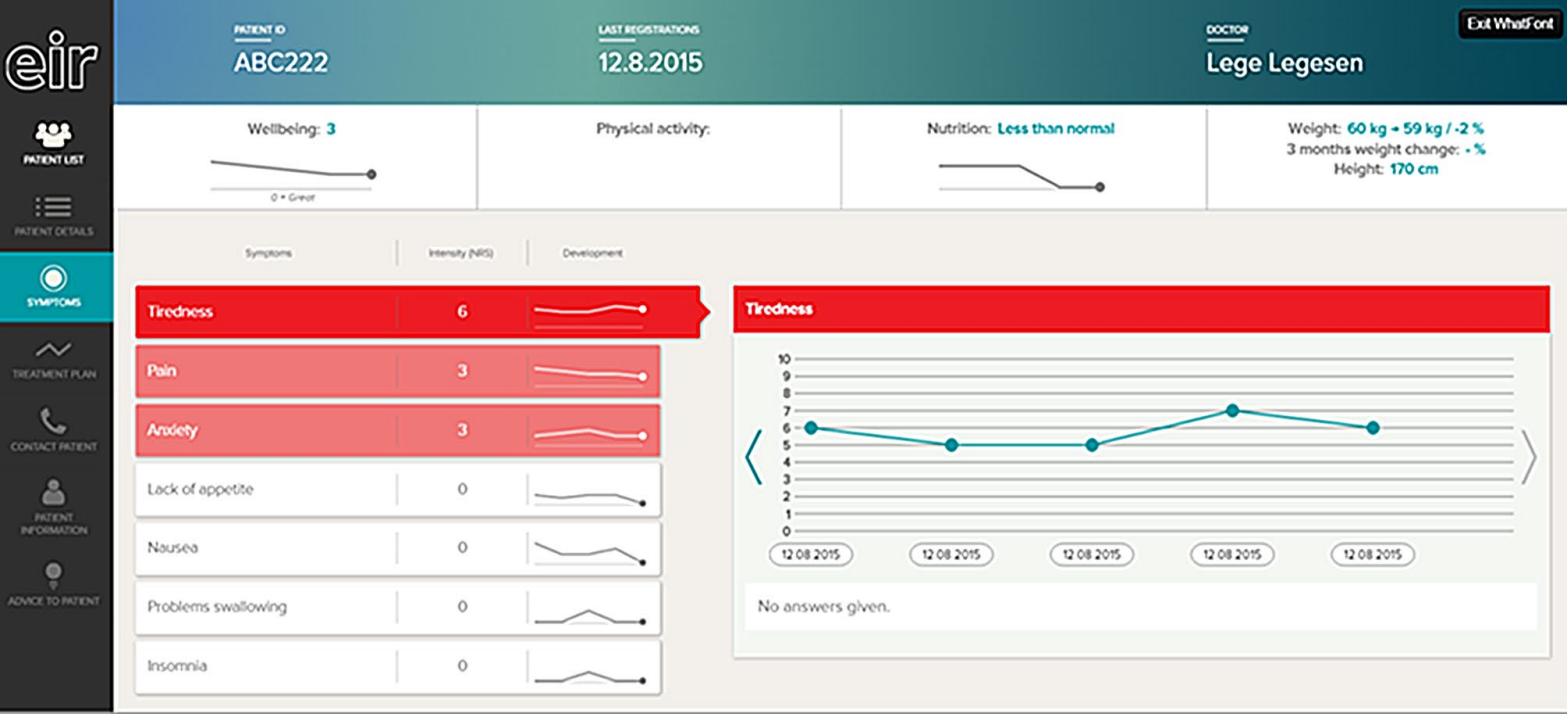
Eir-Doctor: overview. Present symptom intensity to the left, graphical overview of symptom intensity to the right [22]

Eir is a digital tool developed for cancer patients to facilitate systematic symptom assessment in all patient encounters, thereby improving patient-centred care by acknowledging the patient’s voice. Eir’s content is based on international guidelines, well-validated questionnaires, and feedback from clinically experienced experts [22, 23]. Patients indicate their symptoms from a list of the nineteen most frequent symptoms among cancer patients, with follow-up questions for endorsed symptoms. Examples include pain intensity, location, triggers, characterization, and effect of medication, for a patient who marks the pain symptom. The responses, either numerical (0–10) or descriptive, are immediately available to HCPs (see Figs. 1 and 2 [22]). Symptoms are displayed hierarchically using a traffic light method on the scales or with applicable descriptors. Eir also shows symptom development over time and pain locations on a body map. The tool has been used in various clinical studies and routine care settings [21, 24, 25].

### Study setting

Eir was intended as part of a complex intervention in outpatient cancer care facilities in Norway through the nationwide c-RCT PALLiON [21]. Six hospitals were randomized to the intervention arm, and the intervention consisted of three separate parts. One part was the systematic assessment of symptoms in all consultations, preferably using Eir [21]. Each intervention site had an oncologist as the PI and one study nurse.

### Sampling

An invitation to participate was sent to the six intervention sites by a delegate from the PALLiON management core group. Given the nature of the research question, a purposeful sampling strategy was adopted to gain insight into the factors influencing the implementation process. The inclusion criteria were experience as a PI or study nurse at one of the intervention sites in PALLiON (*n* = 13). However, one site declined participation due to time constraints, while the data protection policy prevented implementation at another site. Consent to participate was ultimately obtained from nine HCPs across four sites (*n* = 9). Although the pool of participants was limited, this limitation was inherent to the study’s design and scope [26]. Demographics are presented in Table 1.

**Table 1:** Overview of the study sample

Variables	Study sample	Average (Range)
Gender		
Female	6	
Male	3	
**Profession**		
Oncologist	5	
Cancer Nurse	3	
Palliative Nurse	1	
**Years of experience as HCP**		24 (15–36)
0–5	0	
5–10	0	
10–15	2	
>15	7	

### Data collection

Six individual interviews with PIs and study nurses and one focus group (FG) were conducted between January and November 2021. Due to the Covid-19 pandemic, all interviews were conducted digitally. Based on the aim of the study, a semi-structured interview guide was developed prior to the first interview. The FG followed the same guide as the individual interviews, and the guide evolved during the interview process as new themes emerged that were relevant to the aim (Supplementary Material 1). The participants provided rich and detailed insight into their perceived barriers and facilitators, as well as the impact that implementing Eir at each site had on their working routines. The depth of the data gathered in the interviews allowed for a thorough exploration of the research questions. The individual interviews were conducted by the first author (TSS), who also moderated the FG. The FG was co-moderated by the last author (MHM). All interviews were audiotaped and transcribed verbatim by the first author (TSS). To ensure that anonymity was maintained, all identifiable information was removed from the dataset. Unique codes were allocated to each participant. These codes serve as the primary references to individual responses throughout the article.

### Data analysis

Guided by framework analysis (FA) [27], we analysed the transcribed text using a combination of an inductive and deductive approach. By combining these two approaches, we could rely on ideas arising both from empirical data and existing knowledge [28, 29]. Moreover, it ensured that text fragments not fitting into rigid pre-existing categories were included [30].

To gain insight into the determinants affecting the implementation of Eir, we used the Consolidated Framework for Implementation Research (CFIR) in the deductive stages of the analysis. CFIR is a theoretical, determinant implementation framework consisting of 39 constructs categorized in five multilevel domains: *the innovation* (in this study: Eir), *outer setting* (e.g., study management, and laws and legislation), *inner setting* (e.g. management at the ward, culture), *individuals* (e.g. individuals working at the ward), and *the implementation process* [31–33]. CFIR aims *to predict or explain barriers and facilitators (determinants*,* independent variables) to implementation effectiveness* [33]. In addition, CFIR can be used to retrospectively elucidate the outcome of an implementation [33].

**Fig. 3:**
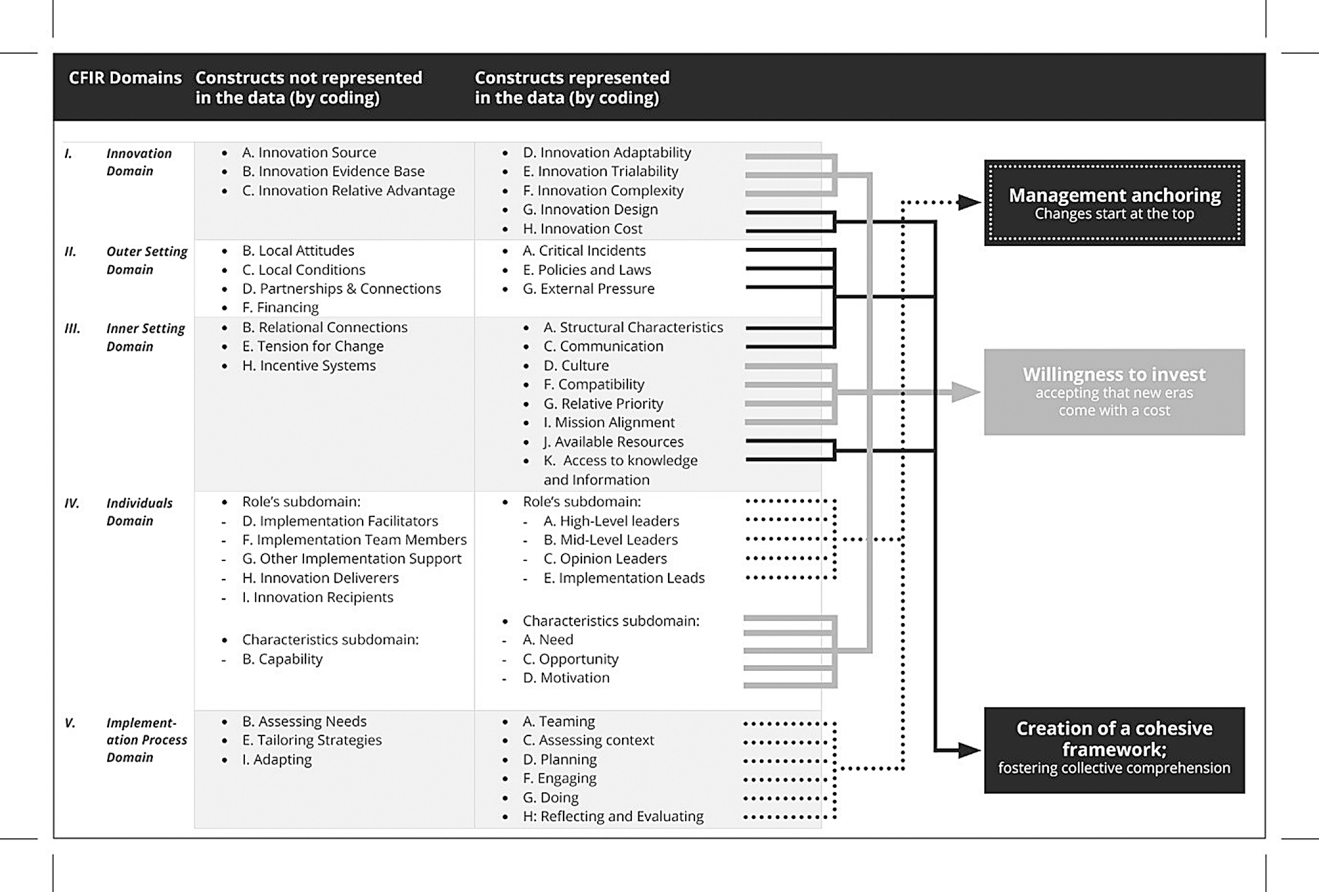
Comprehensive overview of all CFIR domains, including constructs not represented in the data (by coding) and constructs represented in the data (by coding). The figure illustrates the link between the constructs represented in the data and the final themes in the analysis. *Overview of domains and the represented constructs in CFIR (through coding), and the development of themes included in the final analysis

First, we familiarised ourselves with the data by reading and re-reading the transcripts. The first and last author (TSS, MHM) separately read all transcripts and made notes about initial thoughts and ideas. Subsequently, these preliminary perceptions were discussed. Second, the first author (TSS) performed an inductive coding of all interviews. To improve rigour and consistency, all authors discussed and reached consensus regarding the inductive codes for three of the transcripts. In the next, deductive stage of the analysis, an a priori codebook based on CFIR [33] was created. The five domains in CFIR were defined as themes and constructs as sub-themes. Then, we added this to a chart using NVIVO (version 12; QSR International). In the third stage, TSS systematically applied the framework to each interview. Based on discussions with the other team members, adjustments were made. In the fourth stage, the material was reduced into understandable, but brief summaries of the participants statements [27]. Key phrases were highlighted to retain the essence of the raw data. This formed the framework matrices. In the final step, we synthesized data by mapping and interpreting [34]. To enhance rigour, we reviewed charts to see the whole dataset, comparing themes (domains) and sub-themes (constructs) to each other [34]. In the concluding phase of the analysis, subsequent to the identification of intra-case and inter-case linkages and patterns, we formulated three principal themes that spanned across the quintet of domains within CFIR. The use of CFIR ensured a structured and comprehensive analysis, which contributed to a robust understanding of the processes involved in the implementation of Eir (see Fig. 3) [33].

## Results

The descriptions from participants concerned three broad themes: (1) Willingness to invest; accepting that new eras come with a cost, (2) Management anchoring; changes start at the top, and (3) Creation of a cohesive framework; fostering collective comprehension.

### Willingness to invest: accepting that new eras come with a cost

The first theme encapsulates the HCPs’ readiness to devote time and effort to implementing Eir, anticipating future benefits. The findings suggest a disparity between oncologists and nurses regarding the amount of time and effort they were willing to invest to achieve successful implementation. Participants acknowledged that to leverage the benefits of digital symptom management, a transformation in their patient consultation methods would be indispensable. However, there was a divergence in the participants’ views on whether the potential benefits derived from implementing Eir outweighed the associated costs.

Nurses emphasized how Eir facilitated a more comprehensive and patient-centred symptom mapping. In addition, nurses perceived the tool as user-friendly for both patients and HCPs. These experiences influenced nurses’ motivation and served as a significant facilitator throughout the process.

(…) you made this so clear, these scores they did and the description, for example of pain, it became much broader and much more accurate than on an ESAS. So that you kind of got to the bottom of what this pain really is. (N9-nurse)

There is a learning effect in going through Eir because, just describing the pain, all the nuances that then arise, come up, gives them [patients] words that they may not have been so conscious of (N4-nurse).

Conversely, oncologists demonstrated a diminished propensity to allocate time and resources towards the implementation process. Their focus was primarily directed towards the potential benefits and drawbacks that the utilization of Eir could introduce into their own workflow, rather than the improvement of patient symptom treatment. The perception that the adoption of Eir would necessitate alterations to the clinical consultation, coupled with the realization that it demanded additional effort without an apparent clinical advantage, contributed to an amplified resistance towards the tool. Furthermore, oncologists expressed significant concerns about Eir´s inability to integrate with existing hospital systems. They argued that a lack of integration made it impossible to adapt Eir into the current workflow, which in turn strengthened their resistance to its use. One oncologist said:

To have a software with its own username and password, in addition to everything else we have, (…) I believe that if this is to be implemented, it must be integrated into the patient’s medical record, where you can access it [Eir] via a simple key [on the computer]. (N6-oncologist)

Moreover, nurses noted how they, and the patients, spent time and resources on registration without seeing any results in terms of improved symptom management. A nurse explained it like this:

It would be much more organized for the doctor if the doctor had used [Eir], but the doctor doesn’t use it. (…) When it’s not utilized as hoped, it really becomes wasted time. (N2-nurse)

The mismatch between the perceived benefits of using Eir created a gap between nurses and oncologists in terms of the main outcome. Moreover, several oncologists did not find it necessary to modify their symptom management practices. The absence of a joint mission alignment ultimately surfaced as a significant barrier. The trialability of the innovation constituted another considerable barrier. Several participants recounted their initial impression that Eir was to be fully implemented and utilized by all patients. The realization that the innovation was specifically tailored for the study patients and for a limited duration notably affected the oncologists’ inclination to invest in the implementation process.

### Management anchoring: changes start at the top

The degree of management anchoring at different levels had a high impact on the implementation process and was perceived as decisive for the outcome of the implementation. At a local level, the degree of management anchoring was emphasized as either a facilitator or a barrier to the implementation.

Several participants expressed that the intricate nature of the innovation meant that Eir’s implementation necessitated a tightly knit decision-making process across various management levels. This was particularly evident when complex decisions, requiring substantial management authority, came up- a resource that participants felt they lacked. For instance, an oncologist underscored the critical role of top-tier leadership in addressing issues related to financial compensation for ICT services. The lack of sufficient management power prior to the implementation resulted in a significant expenditure of time, effort, and resources to resolve these issues.

No, we had to go through our management and talk to them. The technical director of medicine got involved, even the CEO, so it wasn’t a straightforward matter. (N5 -oncologist)

Furthermore, involvement from mid-level leaders was perceived as a prerequisite for success with the implementation of Eir at each site. Several participants highlighted the lack of such involvement, which affected both their ability to use Eir and their motivation to do so. A nurse said it like this:

No, I don’t feel that they [management] (…) I haven’t heard a word about Eir, actually. Other than what I’ve tried myself, at least I managed to get those [patients] who were included in [the RCT], and that’s where it ended (N2-nurse).

Moreover, the lack of mid-level leader involvement created space for employees unwilling to commit to the implementation. At a local level, this low managerial anchoring led to reduced decision-making authority. This was a major barrier to the implementation of Eir. One oncologist said:

If one were to allocate 10 min consistently each week from the management team, it wouldn’t suffice for a colleague at the same level to encourage their fellow colleagues to work in a certain manner. I consider this to be a directive from management. (…) Because when you’ve received instructions from the boss to do something a certain way, and you don’t comply, you must be accountable (N10- oncologist).

The participants had defined roles in the implementation at the local level. These leads were important facilitators. Both oncologists and nurses considered sufficient time and resources as essential elements for success in this role, but also emphasized that the role was individually demanding in an already hectic everyday work life. A nurse said:

‘Ann’ [the local PI] sent out emails, spoke with some people, and persevered. I was proactive in reminding them that patients [who took Eir] were coming. Especially as time went on and we realized we needed to put in more effort, right? It didn’t happen on its own, so we truly made an effort. And it helped, for some. (N8-nurse)

Some participants experienced that their roles were assigned to them by their leaders, devoid of any chance to influence this decision-making process. This lack of power regarding their own roles led to a sense of reluctance, which in turn negatively affected the implementation process.

The importance of management anchoring also applied in the outer setting, referring to the management group leading the RCT. Some oncologists experienced a lack of pre-defined management anchoring, which was demanding to solve during the implementation process. They called for greater commitment and interaction from study management.

### Creation of a cohesive framework: fostering collective comprehension

The third theme highlights the importance of establishing a cohesive framework in order to succeed with implementing Eir. The results indicate that the implementation of Eir necessitated a pre-establishment of certain structures by the study management. This included collaboration with ICT departments and ensuring the tool’s readiness. Additionally, it was found that other structural elements, such as individual roles, required organization and customization at a local level. The creation of a cohesive framework was described as a critical factor for success, as it fostered a collective comprehension among those involved in Eir’s implementation.

Participants emphasized that they perceived the implementation as being premature. Each site encountered numerous critical incidents at the onset of the implementation process. For instance, several sites faced significant challenges with login procedures during the early stages of implementation. These issues subsequently impacted their ability to utilize Eir. Particularly, major events stemming from technological difficulties were deemed barriers to the implementation process.

Unfortunately, it took a very long time before we got started with Eir, primarily due to issues related to its implementation. (…) Consequently, one becomes a bit frustrated beforehand because it took so long. (N8-nurse)

One oncologist spoke about how the lack of a ready-made tool affected the implementation process:

But in terms of changing clinical practice and being implemented, the prerequisites were not in place before we started, I would say. (N3-oncologist)

Participants considered teaming with ICT personnel as crucial, yet extremely challenging. Several physicians highlighted how a lack of pre-established collaboration with ICT required massive resources to solve. Most oncologists stated that they felt overwhelmed by the workload this triggered.

The collaboration between our ICT supplier and the study management did not go well. There was a lot of resistance all the time, and whenever things didn´t work, we didn´t really receive any assistance (N1-oncologist).

The fact that there was an external server located in [another hospital], and the process of obtaining permission to pass through the ICT security networks and such—it involves committees for this and committees for that. Then there are budget considerations: who should cover the costs of certificate creation, who should pay for this, who should pay for that. And there’s also the matter of emails (N10-oncologist).

Several participants highlighted that data protection and security issues were essential factors throughout the whole process. Both oncologists and nurses reported that they had concerns regarding the tool’s ability to preserve privacy due to how the tool was designed for the study. However, some participants also emphasized that the strict legislation regarding privacy issues hindered new innovations:

I see many valid reasons for the system being so rigid; after all, we handle highly sensitive information. However, this approach doesn’t align well with innovation in other areas—such as implementing new work methods or involving external actors. That´s were conflicts arise. (N10-oncologist)

Neither nurses nor physicians manifested a robust commitment or a sense of obligation to tailor the implementation process to local circumstances. They uniformly attributed the lack of success in implementing Eir at their site to external elements, such as study management or external factors. The absence of local integration and sense of accountability emerged as barriers in the implementation process.

## Discussion

In this qualitative study, we explored the barriers and facilitators involved in the implementation of ePROMs through a c-RCT within the context of hospital-based cancer care. The study unveiled a disparity among HCPs in their willingness to invest time and effort in implementing Eir, presenting divergent views on the cost-benefit ratio. This willingness to invest was significantly influenced by the extent of management anchoring and the establishment of a cohesive framework. Our findings highlight the multifaceted challenges inherent in such transitions, emphasizing the necessity for a meticulous alignment of numerous factors to ensure successful implementation.

First, the willingness of HCPs to invest time and effort in the implementation varied significantly between professions. Nurses perceived the advantages provided by Eir, emphasizing its potential to provide a more comprehensive and patient-centered approach to symptom management. In contrast, oncologists expressed concerns about the alignment of the tool with their established routines and questioned its ability to enhance clinical outcomes.

The “culture” within the healthcare setting plays a critical role in the successful adoption of new innovations [33]. Our findings highlight a dichotomy between the patient-oriented approach adopted by participating nurses and the healthcare provider-centric perspective articulated by the oncologists. This dichotomy underscores a disparity in the degree of commitment levels towards the implementation process across the professional spectrum. This tension eventually manifested as a misalignment, posing a significant impediment throughout the Eir implementation process. The identified gap between different professions could be further elucidated by the diffusion of innovation theory (DOI), which explains how individuals within a social system, such as an outpatient clinic, adopt new innovations in various ways [35]. Furthermore, it could also shed light on the diverse adoption behavior expressed by different professions. Recognizing both the individual and professional influence wielded by those engaged in local-level implementation could be pivotal when implementing ePROMs in a hospital setting. It could also be crucial to address and understand professional disparities both before and during the implementation process to prevent these differences from becoming barriers to implementation.

In a systematic review, Granja, Janssen and Johansen [20] found that workflow was one of the most common factors influencing outcomes of eHealth interventions. This aligns with our findings, which indicate that HCPs reluctance towards Eir evolved as they experienced the tool disrupting established workflows. Resistance to change is a well-known barrier to innovation in healthcare [18, 36]. The utilization of ePROMs enhances patient involvement and empowers patients in their interactions with HCPs [18]. Healthcare is shifting towards a more value-based and patient-centred focus [37, 38]. This shift necessitates patient involvement, shared decision-making, and a holistic approach to ensure a healthcare service rooted in patient-centeredness [39, 40]. Professional identity encompasses behaviors, utility, and attitudes related to being a professional [41]. Recognizing the power shift from HCPs to patients, particularly in the context of professional identity [20, 36], could be crucial for successfully implementing ePROMs.

Furthermore, the trialability of an innovation usually serves as a facilitator in the implementation and adoption of an innovation [33, 35]. In contrast, our findings suggest that HCPs resistance to Eir evolved when they realized that the tool was for a subset of patients and within a limited period only. It seems crucial to involve all clinical stakeholders at an early stage in the process [18, 42], make sure that the information is received by those who will be involved, and provide support in the initial phases of the implementation. This ensures a mutual understanding of the overall objective, and the level of effort required to achieve the goal.

Secondly, management anchoring was vital for the successful implementation of Eir. Sites that lacked sufficient management support described a sense of diminished decision-making power, which posed as a hurdle in the Eir implementation process. The role of both formal and informal leaders is particularly crucial when implementing changes in healthcare, as they can either facilitate or impede the process [36]. Defining roles and responsibilities early in the process might be essential, as it can influence the implementation outcome. Moreover, in accordance with prior research [20], HCPs identified the role of implementation leads as a facilitator in the implementation. However, they also faced challenges at the individual level when executing this role, mainly due to a lack of influence or power when interacting with colleagues. This also impacted their willingness to invest in the implementation. Strengthening the collaboration between management, at both local and outer settings, and those leading the implementation (implementation leads) could enhance decision-making authority, thereby increasing the likelihood of successful implementation.

Third, and strongly related to the willingness of HCPs to invest, was the forming of a cohesive framework. Eir was perceived as being implemented prematurely, which triggered delays and extra workload for those involved. This, in turn, created space for increased resistance. Combined with low managerial anchoring, it was decisive for the outcome of the implementation. Investing time and resources in the pre-implementation stage can be pivotal in preparing an organization for the changes required if one is to succeed with implementing ePROMs [42]. The external focus when barriers occurred is in line with findings from a previous study showing that HCPs tend to explain barriers to optimal patient care with external factors to maintain a positive self-view [43]. Closer collaboration, both at a local and central level, could be factors that facilitate the implementation of ePROMs.

### Limitations

Some limitations in this study must be acknowledged. Firstly, while our study was conducted within the context of Norwegian outpatient cancer clinics, we acknowledge that cultural factors may influence the experiences and perceptions of HCPs. The findings may reflect specific cultural and organizational contexts unique to Norway. Nevertheless, many of the barriers and facilitators identified, such as professional disparities, leader involvement, and technological challenges, are likely to be relevant across different settings. Furthermore, the implementation of ePROMs in oncology may present unique facilitators and barriers compared to other areas due to multiple factors, such as the complexity of care, the patient population, and the emotional and psychological aspects of the disease. This could influence both the integration of ePROMs, how they are utilized and perceived, and the acceptance and effectiveness of such tools. Secondly, we did not assess participants’ general ability to use digital tools, which could potentially influence their perceptions of ePROMs. However, given the participants’ age and extensive experience within healthcare services, it is reasonable to assume they have some familiarity with digital tools and, consequently, a basic level of digital literacy. Thirdly, one could argue that data saturation is difficult to achieve with a small number of participants. However, given the aim of the study, data saturation was not the explicit goal. Instead, we argue that thematic saturation was achieved within the scope of the study. We identified recurring themes and patterns across the collected data, suggesting that additional data would not likely have provided significantly new insights [44]. Lastly, we did not involve leaders at various levels or HCPs without formal roles in the implementation, whose perspectives could have provided additional insight into different facets of the process.

## Conclusion

In conclusion, our study underscores the multifaceted nature of factors that can act as facilitators and/or barriers in the implementation of ePROMs in an outpatient clinic. We emphasize the importance of considering the diverse perspectives across professions, as these differences could potentially emerge as barriers during implementation. Moreover, fostering interprofessional collaboration could be crucial to ensure successful implementation of ePROM tools such as Eir. Our findings also highlight the necessity of transforming workflows to fully leverage the potential advantages of ePROMs. We identified that encouraging HCPs readiness to dedicate the necessary time and resources towards this paradigm shift can be facilitated through robust management anchoring and the establishment of a cohesive framework. Furthermore, our study underscores the importance of acknowledging the perceived professional advantages in the acceptance and integration of new tools within clinical cancer care. Recognizing these advantages is pivotal for the successful incorporation of innovative tools in the realm of cancer care.

Following the insight gained in this study, future research should explore strategies to bridge professional disparities and promote a shared understanding of the value of ePROMs. Furthermore, in-depth research on patient perspectives and experiences regarding the use of ePROMs could facilitate their use among HCPs, thereby paving the way forward for successful implementation of ePROM tools in the future.

## Electronic supplementary material

Below is the link to the electronic supplementary material.

Supplementary Material 1

## Data Availability

The dataset generated and analysed during the current study are not publicly available due to privacy concerns but are available from the corresponding author on reasonable request.

## Abbreviations

CFIR: Consolidated Framework for Implementation Research
c-RCT: Cluster Randomized Trial
ePROMs: Electronic Patient -Reported Outcome Measures
FA: Framework Analyses
FG: Focus Group
HCPs: Health-Care Professionals
PALLiON: Palliative Care Integrated in Oncology
PCC: Patient-Centred Care
PI: Principal Investigator
PROMs: Patient-Reported Outcome Measures
