# Supplementary material for: First-time use of electronic patient-reported outcome measures in a cluster randomized trial: a qualitative study
Source: J Patient Rep Outcomes. 2025 Jan 20;9:9. doi: 10.1186/s41687-025-00840-1 (PMC11746995; doi:10.1186/s41687-025-00840-1)
Supplement: Supplementary file 1 — Supplementary Material 1 [file 41687_2025_840_MOESM1_ESM.docx]

**Supplementary Material 1:** Interview guide.

| **Could you commence by elucidating the rationale behind the decision to implement EIR within your department?** |
| --- |
| **Could you share the experiences you have garnered from the introduction (implementation) of “Eir” within your department?** |
| - Could you expound on any challenges encountered during the establishment of a new electronic tool (Eir)? |
| - What factors, if any, have facilitated the implementation process? Could you provide an example? |
| - What factors, if any, have impeded the implementation process? Could you provide an example? |
| - How do you perceive the use of Eir in comparison to previous paper-based symptom mapping tools? |
| - What are your contemplations regarding the path forward? |
| **Can you share the experiences you have with the utilization of Eir in clinical work?** |
| - What potential positive factors do you observe in using EIR? Could you provide an example? |
| - What potential negative factors do you observe in using EIR? Could you provide an example? |
| - Do you have thoughts about the future in terms of using EIR? |
| - Has it (Eir) had an impact on the workflow within the department? If so, could you specify? |
| **Are there any aspects we have not discussed that you deem important to include in relation to Eir?** |
